# Editorial: Integrating pediatric oral health and general health

**DOI:** 10.3389/fdmed.2026.1977888

**Published:** 2026-09-28

**Authors:** Faith Miaomiao Zheng, Palinee Detsomboonrat, Duangporn Duangthip

**Affiliations:** 1 Faculty of Dentistry, The University of Hong Kong, Hong Kong, Hong Kong SAR, China; 2 Faculty of Dentistry, Chulalongkorn University, Bangkok, Thailand; 3 College of Dentistry, The Ohio State University, Columbus, OH, United States

**Keywords:** general health, healthcare, oral health, pediatric dentistry, psychological wellbeing

Oral health is an integral component of general health and is essential for eating, communication, sleep, psychological wellbeing, education, and social participation. Nevertheless, oral diseases remain among the most prevalent noncommunicable diseases worldwide, affecting approximately 3.5 billion people and disproportionately burdening disadvantaged populations (1, 2). Dental caries is particularly common during childhood and can cause pain, infection, eating difficulties, disturbed sleep, and poorer quality of life for children and their families (3, 4).

Despite this burden, oral health services are frequently separated from primary and pediatric healthcare. This separation can delay risk identification, prevention, referral, and treatment. Evidence suggests that integrating oral health into primary care can improve preventive service delivery, referral pathways, professional collaboration, and access to care, although effective models must be adapted to local health systems and population needs (5, 6). The World Health Organization has therefore identified the integration of essential oral healthcare into primary healthcare as a central objective of its Global Oral Health Action Plan for 2023–2030 (2).

Against this background, the present Research Topic brings together eleven articles examining pediatric oral health through environmental, developmental, behavioral, clinical, technological, and public health perspectives.

Several contributions demonstrate why pediatric oral health assessment should extend beyond the mouth. Dardeer et al. examined second-hand smoke exposure among 427 children in Saudi Arabia. Children exposed to smoke in one, two, or three private settings had progressively higher mean caries scores than unexposed children. Although this cross-sectional study cannot establish causation, its findings support incorporating tobacco exposure assessment and counselling on smoke-free homes and cars into pediatric dental care.

Hajjar et al. explored probable sleep bruxism and the risk of obstructive sleep apnea among 281 Lebanese children in the mixed dentition stage. Probable sleep bruxism was identified in 61.2% of participants, but its association with obstructive sleep apnea risk did not reach statistical significance. Bruxism alone should therefore not be interpreted as a diagnostic indicator of sleep apnea. Nevertheless, dental professionals may contribute to early recognition by asking about snoring, breathing difficulties, sleep quality, and related symptoms before referring children for medical assessment when appropriate.

Carda Navarro et al. reviewed evidence concerning dental occlusion, postural control, and plantar parameters. Only seven studies met their eligibility criteria, and the two involving growing participants reported conflicting findings. The overall certainty of evidence was low to very low, and no consistent pediatric association could be established. This review provides an important reminder that plausible connections between oral and systemic health require rigorous and reproducible evidence before influencing clinical treatment.

The family environment also shapes oral health. Macrì et al. followed 90 mothers during the postpartum period. Although both breastfeeding and formula feeding groups initially improved after receiving oral hygiene education, breastfeeding mothers showed less favorable plaque, gingival bleeding, and microbiological findings at six months. These results should not be interpreted as questioning the established benefits of breastfeeding. Rather, they indicate that breastfeeding mothers may require additional oral health support during a demanding period of physical and psychological adjustment. Integrating maternal dental monitoring into perinatal care could benefit mothers while supporting infant health.

The importance of population surveillance is illustrated by Sun et al., who analyzed data from all 2,834 children attending kindergartens, primary schools, and middle schools in Feng'an Town in southern China. Caries affected 79% of kindergarten children, 88.5% of primary school children when primary and permanent teeth were considered together, and 75.9% of middle school children in their permanent dentition. The substantial burden supports coordinated prevention involving families, schools, healthcare services, and local authorities. Potential measures include parental education, fluoride programs, timely dental assessment, and minimally invasive treatment.

Clinical indices alone, however, cannot fully describe the effects of oral disease. Yu et al. translated and culturally adapted the Scale of Oral Health Outcomes for five-year-old children into Mandarin. The instrument showed good internal consistency and construct validity in 259 child and parent pairs. The parental version distinguished between children with and without caries, whereas the discriminant validity of the child version was limited. This instrument can complement clinical assessment by capturing how oral conditions affect eating, speaking, playing, sleeping, smiling, and self-confidence.

Phyo et al. cross-culturally adapted an oral health literacy questionnaire for Myanmar migrant mothers of preschool children in Thailand. Among 133 mothers, the questionnaire demonstrated acceptable internal consistency and a two-factor structure, but test-retest reliability was poor, and total scores did not differ significantly by children's caries experience or mothers' previous dental visits. The instrument therefore offers a preliminary basis for identifying oral health literacy needs in this underserved population, but further validation is required before broader use.

Equity and inclusion are further addressed by Bainazarova et al. Their systematic review and meta-analysis included 24 studies comparing children with Down syndrome or autism spectrum disorder with neurotypical peers. The pooled results showed no consistent differences in plaque, gingival, oral hygiene, or caries indices, largely because of substantial heterogeneity and low certainty of evidence. These inconclusive findings do not imply that the groups have equivalent experiences of care. Communication, sensory, behavioral, financial, and access barriers remain important. Inclusive preventive programs, professional training, and individualized care are therefore essential.

Three contributions focus on treatment and children's experiences of dental care. Joshi et al. reviewed five studies concerning stainless steel crowns for permanent molars severely affected by molar incisor hypomineralization. These crowns generally demonstrated higher survival rates, better retention and marginal integrity, and a reduced need for retreatment compared with alternative restorations during follow-up periods of 12 to 24 months. Although stronger trials with longer follow-up are needed, stainless steel crowns appear to provide a durable and economical option for severe cases.

Ra'ed-Naser et al. reviewed virtual reality as a behavioral management method during pediatric dental visits. Virtual reality generally reduced anxiety and improved cooperation, with immersive audiovisual distraction producing the most favorable responses. However, variation among studies and substantial risks of performance and measurement bias limit the certainty of some outcomes. Virtual reality should therefore be considered a useful adjunct selected according to the child's age, preferences, sensory profile, and anxiety, rather than a replacement for communication and established behavior guidance.

Hu and Song examined pain trajectories in 200 children with recurrent aphthous stomatitis. They identified three patterns: high pain followed by relief, moderate pain followed by improvement, and persistently low pain. Ulcer size, number, type, attack frequency, and picky eating were associated with trajectory membership. These findings support repeated pain assessment and individualized follow-up. However, prospective studies are required before nutritional or therapeutic interventions can be recommended based on these associations.

Together, these articles demonstrate that pediatric oral health cannot be separated from general health or the environments in which children live. Tobacco exposure, sleep, nutrition, neurodevelopment, pain, anxiety, maternal health, and technology all influence how oral conditions are understood and managed. The Research Topic also reveals important evidence gaps, including heterogeneous methods, observational designs, limited longitudinal evaluation, and insufficient representation of diverse populations.

Integration therefore requires more than collaboration in principle. It calls for shared risk assessment, clear referral pathways, inclusive preventive services, and outcomes that reflect both clinical disease and children's quality of life. By connecting dentistry with pediatrics, perinatal care, public health, schools, and families, we can move toward earlier prevention, more equitable access, and more responsive care for every child.
